# Low birthweight of children is positively associated with mother’s prenatal tobacco smoke exposure in Shanghai: a cross-sectional study

**DOI:** 10.1186/s12884-020-03307-x

**Published:** 2020-10-08

**Authors:** Ruiping Wang, Ting Sun, Qiong Yang, Qing Yang, Jian Wang, Huan Li, Yue Tang, Liang Yang, Jie Sun

**Affiliations:** 1 Office of Clinical Research Center, Yueyang Hospital of Integrated Traditional Chinese and Western Medicine, Shanghai University of Traditional Chinese Medicine, 110 GanHe Road, Hongkou District, Shanghai, 200437 China; 2 Songjiang Maternal and Child Health-care Hospital, Shanghai, China; 3 Songjiang Fang Song Community Health Service Center, Shanghai, China; 4 Jing’an Institute of Maternal and Child Health-care, Shanghai, China

**Keywords:** Prenatal tobacco smoke exposure, First-hand smoke, Second-hand smoke, Low birth weight, Tobacco control

## Abstract

**Introduction:**

Low birthweight (LBW) is a significant public health issue, and maternal smoking is the most prevalent preventable cause of LBW. But there is limited evidence on association of LBW among children and cigarette smoke exposure in mothers in China. In this cross-sectional study, we try to explore if the LBW in children is positively associated with mothers’ prenatal cigarette smoke exposure.

**Methods:**

We selected 8, 586 mothers and their singleton children in 2018 in Songjiang district, Shanghai. Birthweight of children and gestational weeks of mother was identified by birth records in the hospital, we classified mothers’ prenatal cigarette smoke status into the first-hand smoke (FHS) exposure and the second-hand smoke (SHS) exposure. We use SAS 9.1.3 software to calculate the prevalence of children’s LBW and the prevalence of mothers’ prenatal cigarette smoke exposure including FHS and SHS. Chi-square test and logistic regression were used to analyze the difference.

**Results:**

In 8, 586 women, The prenatal FHS and SHS exposure prevalence was 0.9 and 20.8%, respectively. The mean birthweight of children was 3315.5 g with a standard deviation of 497.2 g, the mean birthweight was 167.7 g and 66.1 g lower in children born to mothers with prenatally FHS and SHS exposure compared with those children whose mother were not exposed, respectively. The children’s LBW prevalence was 4.7% in this study. By comparing with children whose mother were not exposed, the LBW prevalence was higher among children whose mother were prenatally exposed to FHS [OR (Odds Ratios) = 2.91, 95% confidence interval (CI) (1.49, 5.68)], and SHS [OR = 2.35, 95% CI (1.90, 2.89)].

**Conclusions:**

Children’s LBW is positively associated with mothers’ prenatal tobacco smoke exposure both for FHS and SHS. So implementing tobacco control measures is crucial to lower smoking prevalence among women, and decrease smoking prevalence of their family members as well as work fellows.

## Introduction

The birthweight less than 2500 g is defined as Low birthweight (LBW), LBW continues to be a significant global public health issue [1]. It is estimated that LBW occurs in almost 15% of all newborns, and approximately 20 million infants worldwide are born low birthweight each year [2, 3]. Four million newborns die within the first 4 weeks of life each year and low birthweight is a significant indirect cause of these deaths [3, 4]. Due to low birthweight has both short-term and long-term adverse consequences, a global target has been set to reduce this figure by 30% by the year of 2025 [3]. Generally low birthweight can increase the risk of newborn death from a variety of nutritional, metabolic, and infectious processes, and low birthweight is also considered as a marker for newborns at high risk for later neurological, neuropsychological, and psychiatric problems [4–7]. Low birth weight has been associated with a variety of psychiatric outcomes and cognitive disorders in children, including internalizing, attention and social problems, speech and language, and learning disabilities [7, 8]. Therefore, strategies to reduce the frequency of low birthweight could have a measurable impact on the future health condition of children.

Cigarette smoking in childbearing aged women hand its associated adverse outcomes have been a common concern since the 1960s [9]. It is well documented that low birthweight is associated with tobacco use, malnutrition in gestation, sexual transmitted diseases and other maternal infections, adolescent childbearing, etc. [3, 4, 10]. And prenatal tobacco smoke exposure among women is the most prevalent preventable cause of neonatal morbidity, preterm delivery, and low birthweight as well [11]. In western countries, tobacco smoking has been well established as the leading cause of low birthweight in newborns [12], and most primary studies demonstrate the positive associate of smoking in mother and low birthweight in children, but the findings are varied due to population and methodological differences [9]. A recent meta-analysis indicates that maternal exposure to second-hand smoke during pregnancy reduces the mean birthweight by 31–60 g and may increases the risk of low birth weight by 1.16–1.60 times [13, 14], but Goel P [15] reported that the mean birthweight of infants born to mothers exposed to second hand smoke was 138 g less than that of newborns whose mothers’ were not prenatally exposed [16]. Meanwhile, a study in Brazil with a study population of 5166 newborns demonstrates that children with mothers who smoked during pregnancy had a birthweight 142 g lower than those children with non-smoking mothers, and the odds ratio (OR) for LBW among children of smokers was 1.59 [17]. All of these findings presented above stimulate a need to explore the magnitude of correlation between mothers’ prenatal cigarette smoke exposure and children’s LBW with a larger study population size, both for first-hand smoke (FHS) and second-hand smoke (SHS).

China is the largest tobacco producer and consumer in the world, the Global Adult Survey conducted in 2010 indicates that there are 300 million adult smokers in China, and 1 million deaths each year are due to tobacco consumption [18–20]. The active smoking prevalence among pregnant women is estimated to be approximately 25% worldwide [21], in China, the prevalence of active smoking (first hand smoking) among women with pregnancy is lower, ranges from 0.7 to 1.6%, but the prevalence of second-hand smoke exposure among non-smoking women is ranged from 38.9 to 75.1% [22]. Moreover, a cross-sectional study estimating the urinary cotinine concentration in pregnant women in China demonstrates that the prevalence of urinary cotinine detection reaches 87%, indicating that the prenatal second hand smoke exposure among pregnant women are very high [23], but evidence is still limited in China on the association of low birthweight in children and cigarette smoke exposure in pregnant women, both for SHS and FHS.

In this paper, we conducted a cross-sectional study in Songjiang district, a rural area of Shanghai, China. We aim to learn the prevalence of prenatal cigarette smoke exposure among mothers, both for FHS and SHS, and the prevalence of low birthweight in children, and to explore if prenatal cigarette smoke exposure among mother is positively related with the low birthweight among children.

## Methods

### Study population

We conducted this study during March and September in 2018 in Songjiang district of Shanghai. PASS software was applied for sample size calculation, we assumed that the prevalence of low birthweight was 5% in Songjiang district, considering an inspection level (α) of 0.05 and a permissible error (δ) of 0.005 (10% of LBW prevalence), at least 7299 children should be sampled and recruited. Take population size of kindergarten into consideration, we finally selected 20 kindergartens in this study. We applied a cluster sampling method to select study population. First, we randomly selected 20 kindergartens out of all 115 kindergartens in Songjiang District. Second, we recruited all singleton children and their mothers from the 20 selected kindergartens. Overall, a total number of 8, 626 children as singleton and their mothers were recruited in this study. Finally, 8, 586 pairs of mothers and children completed the questionnaire interview and were included in the final analysis. Songjiang Maternal and Child Health-care Hospital Institution Review Board authorized the ethic approval of this study (IRB#20171203), and ahead of the questionnaire interview, research coordinators orally communicated with each mother and their children, and then signed the informed consent papers.

### Data collection

In this study, questionnaire for data collection covered 4 parts (see supplementary file 1). Part one included 8 demographic questions (age and education of mother, family yearly income, residency status, gender of children, etc.). Part two included 10 questions of cigarette smoke exposure during pregnancy, gestation and delivery questions (e.g., *‘Have you ever smoked at least one cigarette every day for over six months during your pregnancy?’, ‘Does anyone smoke around you in workplace or at home for at least 10 minutes each day during pregnancy?’,‘which way is your child being delivered, vaginal or cesarean?’, ‘Is your child delivered before 37 gestational week?’, ‘what is your body weight and height before pregnancy?’, ‘what is the birthweight of your child*? *’, ‘have you been diagnosed as gestational diabetes mellitus (GDM) during the pregnancy? ’* etc.). Part three covered 7 questions which were extracted from the birth records of the mothers and singletons to verify the self-reported information (body weight and body height before pregnancy, children’s gestation week at delivery, the birthweight of children, GDM of mother record during pregnancy, tobacco smoking information, etc.). Part four included information for follow-up contact of mothers and their children.

### Definition and index calculation

In this study, prenatal first-hand smoke exposure (FHS) was defined as a mother who smoked at least one cigarette each day for over 6 months before and during pregnancy [18, 19], and the prenatal second-hand smoke exposure (SHS) was defined as a non-smoking women who exposed to cigarette smoke for at least 10 min each day during pregnancy [22]. We calculated the prenatal FHS exposure prevalence as the number of mother with prenatal FHS exposure divided by the total number of mother, and the prenatal SHS exposure prevalence as the number of mother with prenatally SHS exposure divided by the total number of mother. We defined the low birthweight (LBW) as a newborn weighing < 2500 g at birth, and preterm birth as a baby born alive before *gestational* weeks of 37. In this study, mother’s age was divided into four groups (‘19–25’, ‘26–30’, ‘31–35’, and ‘36–48’). We recorded mothers’ education as completed schooling years and classified it into 3 categories including primary school or illiterate (0–6 year), school of junior high (7–9 years), school of senior high (10–12 years), and college and above (> 12 years). Family yearly income (CNY) was categorized into 4 categories (‘< 50,000′, ‘50,000–10,000′, ‘100,001–150,000′, ‘150,001–300,000′ and ‘> 300,000′).

### Data analysis

We performed the data analysis by SAS software (version 9.1.3). In this study, data was described as frequency and (or) prevalence for qualitative variables and means as well as standard deviations (SD) or median as well as interquartile range (IQR) for quantitative variables. Chi-square test was applied to examine the difference of prevalence of prenatal FHS and SHS exposure among mothers with different demographic characteristics, and student’s t test or Wilcoxon Rank Sum test were applied to examine children’s birthweight difference between mothers with and without prenatal cigarette smoke exposure, and we also employed Chi-square test to examine the prevalence difference of low birthweight (LBW) as well as preterm birth in children between mothers with and without prenatal tobacco smoke exposure. We applied univariable and multivariable logistic regression to calculate the odds ratios (OR) and 95% confidence interval (95% CI) of the LBW prevalence in children between whose mothers with prenatal cigarette smoke exposure and whose mothers without, and to explore the association of prenatal tobacco smoke exposure among mothers and LBW prevalence in children. Directed acyclic graphs (DAGs) method, a graphical tool which provide a way to visually represent and better understand the key concepts of exposure, outcome, confounding and bias, was applied to identify potential confounders in this study, and the confirmed confounders were then adjusted in multivariable logistic regression analysis.

## Results

In this study, mothers’ age ranged from 19 to 48 with an average age of (32.2 ± 3.9) years old, the majority of mothers (71.1%) had an education over college, approximately 50% of mothers had family income over 300, 000 CNY by year, and 52.9% of mothers were local residents. Ahead of pregnancy, 61.5% of mothers had a normal body weight, 17.4% of them were overweight or obesity. 6.2% of mothers were diagnosed as gestational diabetes mellitus (GDM) in pregnancy.

### Prevalence of prenatal cigarette smoke exposure

The prevalence of prenatal FHS exposure among 8, 586 mothers was 0.9%. The prenatal FHS exposure prevalence among mothers of local residents (1.2%) was significantly higher than mother of no-local residents (0.6%), and the prevalence of prenatal FHS exposure was higher in mother with GDM (1.9%) than mothers without (0.9%). Mothers had higher prevalence of prenatal FHS exposure with age over 36 years (1.3%) or under 25 years (2.5%). And the prevalence of prenatal FHS exposure among mothers with education of college and above was lower than mothers with education of illiterate, primary school, school of junior high and senior high. Table 1.

**Table 1:** The demographic feature of pregnant women with first-hand smoke (FHS) and second hand smoke (SHS) exposure in a rural area of Shanghai in 2018, China (*n* = 8586)

Variables	Pregnant women (8586)		FHS exposure (81)		SHS exposure (1785)	
	n	Proportion (%)	n	Prevalence (%)	n	Prevalence (%)
Age (years)^†‡^						
19–25	237	2.76	6	2.53	62	26.16
26–30	2870	33.43	25	0.87	728	25.37
31–35	3858	44.93	29	0.75	730	18.92
36–48	1621	18.88	21	1.30	265	16.35
BMI before the pregnancy ^‡^						
Less than 18.50	1819	21.19	18	0.99	390	21.44
18.50–23.99	5278	61.47	50	0.95	1054	19.97
24.00–27.00	526	6.13	4	0.76	103	19.58
Over 27.00	963	11.22	9	0.93	238	24.71
Education^†‡^						
Illiterate/Primary	71	0.83	1	1.41	14	19.72
Junior High	795	9.26	14	1.76	181	22.77
Senior High	1613	18.79	25	1.55	373	23.12
College and above	6107	71.13	41	0.67	1217	19.93
Family yearly income (CNY)^‡^						
Less than 50, 000	1344	15.65	15	1.12	334	24.85
50, 000–100, 000	1206	14.05	9	0.75	273	22.64
100, 001–150, 000	1117	13.01	12	1.07	239	21.40
150, 001–300, 000	1169	13.62	11	0.94	204	17.45
Over 300, 000	3750	43.68	34	0.91	735	19.60
Residency status^†‡^						
Local resident	4545	52.94	56	1.23	1094	24.07
Non-local resident	4041	47.06	25	0.62	691	17.10
Gestational diabetes mellitus (GDM)^†‡^						
Yes	533	6.21	10	1.88	144	27.02
No	8053	93.79	71	0.88	1641	20.38

^†^the differences between group on prevalence of prenatal first-hand smoke exposure was statistically significant (*P* < 0.01)

^‡^the differences between group on prevalence of prenatal second-hand smoke exposure was statistically significant (*P* < 0.01)

In this study, 1, 785 out of 8, 586 women exposed to SHS prenatally with a prevalence of 20.8%. The prenatal SHS exposure prevalence in mothers aged < 30 years (25.4%) was higher than those aged over 30 years (18.2%). Mothers with a BMI < 18.5 or > 27 had higher prevalence of prenatal second-hand smoke exposure. Mothers with education of junior high or senior high schools were highly exposed to second-hand smoke prenatally than those with an education of illiterate, primary, as well as college and above. Mothers with local residency (24.1%) had higher prenatal second-hand smoke exposure prevalence than mothers without local residency status (17.1%), and the prenatal second-hand smoke exposure prevalence in mothers with GDM (27.0%) was higher than mothers without GDM (20.4%). Table 1.

### Prevalence of low birthweight (LBW) in children

**Fig. 1:**
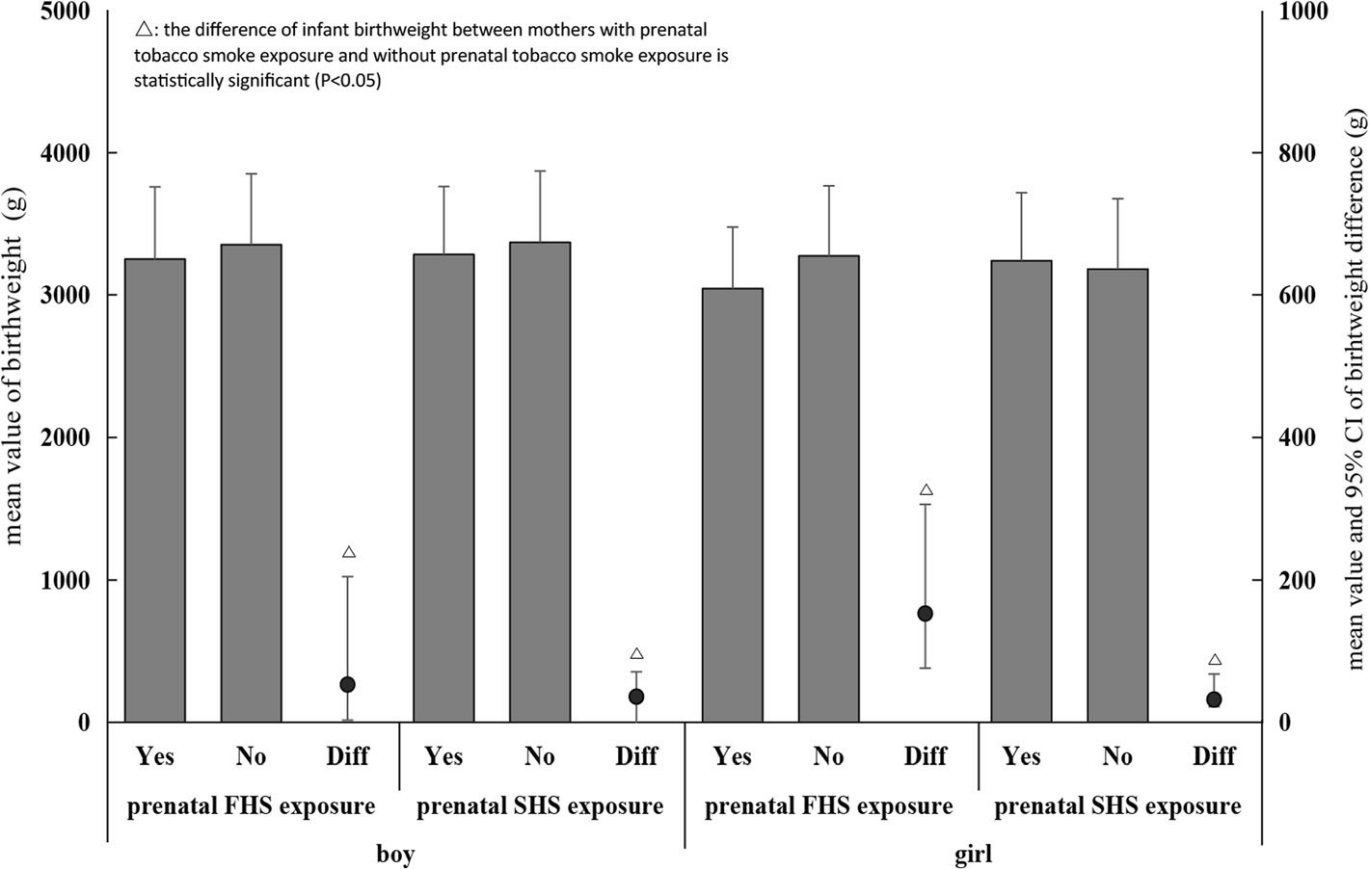
The means of birthweight in children, and the difference of birthweight between whose mothers were with prenatal tobacco smoke exposure and those whose mothers were without prenatal tobacco smoke exposure, both for boys and girls in the year of 2018 in Shanghai China

In this study, 8, 586 singleton children included 4, 595 boys (53.5%). The average birthweight of children born to mothers with prenatal FHS exposure was lower than children born to mothers without prenatal FHS exposure, the difference was statistically significant [D-value = − 167.70 g, 95% CI (− 276.50 g, − 58.97 g)]. Likewise, a lower average birthweight was identified among children whose mothers exposed to SHS prenatally compared with whose mothers without prenatal SHS exposure [D-value = − 66.07 g, 95% CI (− 91.95 g, − 40.18 g)]. Meanwhile, both in boys and girls, the average birthweight of children whose mothers were prenatally exposed to tobacco smoke was significantly lower than children whose mothers were not prenatally exposed tobacco smoke. Table 2 and Fig. 1.

**Table 2:** The birth weight, birth week and gender of children with their mothers prenatally exposed to FHS (first hand smoke) and SHS (second hand smoke) in a rural area of Shanghai in the year of 2018, China

Variables for the children	Total condition	Mother with prenatal FHS exposure		Mother with prenatal SHS exposure	
		Yes (*n* = 81)	No (*n* = 8505)	Yes (*n* = 1785)	No (*n* = 6801)
Birth weight (g), mean (SD)^†‡^	3315.52 (497.16)	3149.38 (481.03)	3317.10 (497.08)	3263.18 (479.23)	3329.25 (500.89)
Birth weight (g), median (IQR)^†‡^	3350 (3000–3650)	3150 (2950–3500)	3350 (3000–3650)	3300 (3000–3570)	3350 (3000–3650)
Birth weight, n (%)^†‡^					
<2500 g	403 (4.69)	10 (12.35)	393 (4.62)	149 (8.35)	254 (3.73)
≥ 2500 g	8183 (95.31)	71 (87.65)	8112 (95.38)	1636 (91.65)	6547 (96.27)
Birth week, mean (SD)	38.61 (2.39)	38.77 (2.43)	38.61 (2.39)	38.62 (2.06)	38.61 (2.47)
Birth week, median (IQR)	39 (38–40)	39 (38–40)	39 (38–40)	39 (38–40)	39 (38–40)
Birth week, n (%)					
<37 weeks	850 (9.90)	9 (11.11)	841 (9.89)	174 (9.75)	676 (9.94)
≥ 37 weeks	7736 (90.10)	72 (88.89)	7664 (90.11)	1611 (90.25)	6125 (90.06)
Gender of the children					
boy	4595 (53.52)	41 (50.62)	4554 (53.54)	931 (52.16)	3664 (53.87)
girl	3991 (46.48)	40 (49.38)	3951 (46.46)	854 (47.84)	3137 (46.13)

*SD* standard deviation

*IQR* interquartile range

^†^the differences in birth weight of the children between mothers with prenatal FHS exposure and without prenatal FHS exposure was statistically significant (*P* < 0.01)

^‡^the differences in birth weight of the children between mothers with prenatal SHS exposure and without prenatal SHS exposure was statistically significant (*P* < 0.01)

The LBW prevalence in children was 4.7% (403/8, 586) in this study. Children born to mothers with prenatal FHS exposure had significantly higher prevalence of LBW than children whose mothers were not prenatally exposed (12.4% vs 4.6%). Similarly, the prevalence of LBW was significant higher in children whose mothers with prenatal SHS exposure than children born to mothers without prenatal SHS exposure (8.4% vs 3.7%). Table 2.

Table 2 indicated that the prevalence of preterm birth was 9.9% (850/8, 586), but there was no significant difference in prevalence of preterm birth between children whose mothers were prenatally exposed to tobacco smoke and children whose mothers were not prenatally exposed to cigarette smoke, both for FHS and SHS. Table 2.

### Association between prenatal cigarette smoke exposure in mother and LBW in children

In this study, we identified that mothers’ prenatal tobacco smoke exposure could increase the LBW occurrence in children. Children born to mothers with prenatal FHS exposure had significantly higher prevalence of LBW than children born to mothers without prenatal FHS exposure [OR = 2.91, 95% CI (1.49, 5.68)], even after the adjustment of potential confounders [OR = 2.77, 95% CI (1.39, 5.53)]. Likewise, Children born to mothers with prenatal SHS exposure also had higher prevalence of LBW than children born to mothers without prenatally exposed to SHS [OR = 2.35, 95% CI (1.90, 2.89)], even with the adjustment of potential confounders [OR = 2.42, 95% CI (1.95, 3.00)]. Table 3.

**Table 3:** The association of mothers’ prenatal tobacco smoke exposure and the prevalence of low birth weight (birth weight less than 2500 g) of children in a rural area of Shanghai in the year of 2018, China

Variables	Low Birth Weight (LBW)		LR(a)		LR(b)		LR(c)	
	Yes n (%)	No n (%)	OR	OR 95%CI	OR	OR 95%CI	OR	OR 95%CI
Prenatal exposure to FHS								
Yes	10 (12.35)	71 (87.65)	2.91	1.49–5.68	***2.77***	***1.39–5.53***	–	–
No	393 (4.62)	8112 (95.38)	1.00	–	***1.00***	***–***	–	–
Prenatal exposure to SHS								
Yes	149 (8.35)	1636 (91.65)	2.35	1.90–2.89	–	–	***2.42***	***1.95–3.00***
No	254 (3.73)	6547 (96.27)	1.00	–	–	–	***1.00***	***–***
Birth week								
<37 weeks	115 (13.53)	735 (86.47)	4.05	3.22–5.09	3.94	3.13–4.97	3.97	3.14–5.02
≥ 37 weeks	288 (3.72)	7448 (96.28)	1.00	–	1.00	–	1.00	–
Age (years)								
19–25	10 (4.22)	227 (95.78)	1.08	0.56–2.08	0.97	0.49–1.91	0.97	0.49–1.88
26–30	113 (3.94)	2757 (96.06)	1.00	–	1.00	–	1.00	–
31–35	211 (5.47)	3647 (94.53)	1.41	1.12–1.78	1.48	1.17–1.88	1.59	1.25–2.02
36–48	69 (4.26)	1552 (95.74)	1.09	0.80–1.47	1.01	0.74–1.38	1.15	0.84–1.58
BMI before the pregnancy								
Less than 18.50	94 (5.17)	1725 (94.83)	1.27	0.99–1.62	–	–	1.28	0.99–1.65
18.50–23.99	218 (4.13)	5060 (95.87)	1.00	–	–	–	1.00	–
24.00–27.00	31 (5.89)	495 (94.11)	1.45	0.99–2.14	–	–	1.23	0.82–1.84
Over 27.00	60 (6.23)	903 (93.77)	1.54	1.15–2.07	–	–	1.33	0.98–1.80
Education								
Illiterate/Primary	5 (7.04)	66 (92.96)	1.68	0.67–4.20	1.62	0.64–4.16	1.55	0.59–4.02
Junior High	57 (7.17)	738 (92.83)	1.71	1.27–2.30	1.77	1.30–2.41	1.75	1.28–2.38
Senior High	77 (4.77)	1536 (95.23)	1.11	0.86–1.44	1.13	0.87–1.48	1.11	0.85–1.46
College and above	264 (4.32)	5843 (95.68)	1.00	–	1.00	–	1.00	–
Gestational diabetes mellitus								
Yes	39 (7.32)	494 (92.68)	1.67	1.18–2.35	1.47	1.03–2.08	1.38	0.97–1.97
No	364 (4.52)	7689 (95.48)	1.00	–	1.00	–	1.00	–

LR(a): Uni-viariate logistic regression

LR(b): Multi-viariate logistic regression to explore the association between LBW of children and prenatal FHS exposure among their mothers, with covariates adjustment of preterm birth, age of mother, education of mother and gestational diabetes mellitus (covariates adjusted during the logistic regression were selected by Directed Acyclic Graph (DAG) method)

LR(c): Multi-viariate logistic regression to explore the association between LBW of children and prenatal SHS exposure among their mothers, with covariates adjustment of preterm birth, age of mother, education of mother, BMI before pregnancy and gestational diabetes mellitus (covariates adjusted during the logistic regression were selected by Directed Acyclic Graph (DAG) method)

## Discussion

It is well documented that tobacco smoke can lead to lung and other cancers, heart diseases, stroke and many other diseases [24], and tobacco smoke includes several harmful chemical substance and is one of the most ubiquitous environmental health hazards both for children and adults [25]. Tobacco smoke exposure during pregnancy is associated with still birth, lower birthweight, higher risk of preterm, and decreased head circumference of newborns at birth [26, 27]. According to the institute for Health Metrics and Evaluation, the prevalence of tobacco smoking in women is 21.9% in Italy, 14.3% in US, 20.3% in UK, and exceeds 25% in France and Belgium [28, 29]. In United States, 8.4% of women were with tobacco smoke exposure during pregnancy [29]. In this study, the prevalence of prenatal FHS exposure among women was 0.9%, which was lower than outcomes reported from the global adult tobacco survey (GATS) [22] in the year of 2012 and SSACB cohort in the year of 2017 [18–20], and some western countries in 2012 [28, 29]. Meanwhile, the prenatal SHS exposure prevalence among women was 20.8%, which was lower than findings in Henan Province (60%), Sichuan Province (70%) and GATS survey in China (53%) in 2012 and an investigation covering 193 countries (40%) [22, 30–32]. The relatively lower prevalence of prenatal FHS and SHS exposure might attribute to the implementation of Smoke-Free environment advocacy and health promotion in Shanghai [33]. Meanwhile, much more residents in Shanghai have noticed the adverse physical effect of tobacco smoke in recent years and finally quit smoking. Nevertheless, we still should pay attention to the high prevalence of prenatal SHS exposure among women under 30 years old, local residents, and women with education of high school (both of junior high and senior high), and tobacco control measures should be implemented mainly in their working and living environment.

Birthweight is an easily obtained measure of fetal development, and low birthweight interferes with newborn survival and negatively affects both childhood and adult life [8]. In this study, the mean birthweight of children born to mothers with prenatal FHS exposure was 167 g lower than children born to mothers without prenatal FHS exposure, meanwhile, the mean birthweight of children whose mothers with prenatal SHS exposure was 66 g lower than those whose mothers were not prenatally exposed to SHS, this was consistent with previous studies [12, 17, 34]. The prevalence of low birthweight among children was 4.69% in this study, which was in line with the developed countries (4–7%), but lower than some developing countries (15%) [21], this could attributed to the excellent maternal healthcare service in Shanghai in recent years. In Shanghai, the proportion of birth taking place in hospital has maintained over 99% since the year of 2005, and 100% of the pregnant women have over 4 times of prenatal hospital visit since the first trimester. In each prenatal hospital visit, obstetricians will provide healthcare service to monitor the fetal development, the gestational condition of mother, and give physical exercise, nutrition and dietary suggestions [35]. The lower prevalence of LBW in children could also be partially explained by the decreased tobacco smoking prevalence in Shanghai [33].

Previous studies demonstrate that low birthweight is associated with social and cultural issues, preterm birth, tobacco use, malnutrition in gestation, sexual transmitted diseases and other maternal infections, as well as maternal lifestyle choices [2–4]. In this study, we demonstrated a significantly higher LBW prevalence among children with preterm birth (13.53%), this was consistent with previous studies [9, 24, 34]. We also noticed that mothers with lower education level were pone to give birth to low birthweight baby, this could be the case that low social-economic status has a direct effect on proper nutrition, maternal access to medical care, and stress level [36]. In this study, we discovered that the prevalence of LBW was higher in children whose mothers were with GDM than those without, but the potential causal association and pathogenic mechanism is still unclear, which need to be clarified in future studies [37].

In this study, we noticed an elevated prevalence of low birthweight among children whose mothers were prenatally exposed to FHS or SHS. The LBW prevalence was 2.91 times higher among children whose mothers were with prenatal FHS exposure, and 2.35 times higher among children born to mothers with prenatal SHS exposure. According to previous studies, the association between mothers’ prenatal tobacco smoke exposure and LBW in children was confounded by preterm birth, GDM of mother, social-economic status, as well as pre-pregnancy overweight [36–38]. In order to address this, the directed acyclic graphs [18] was applied to ascertain these potential confounders. Logistic regression analysis with co-variable adjustment indicated that children still had higher OR value of LBW prevalence if their mother were prenatally exposed to cigarette smoke, the OR values were 2.77 and 2.42 respectively for FHS and SHS. The potential mechanisms underlying the association of LBW with maternal smoking during pregnancy might be explained by the potent toxic constituents in tobacco smoke. Nicotine as a tobacco smoke toxin can cause reduction in uteroplacental circulation, leading to lower maternal weight gain, negative fetal outcomes such as short stature, birthweight loss, and compromised fetal neurological development [34–38].

This study is the first attempt in China to estimate the association of LBW in children and prenatal FHS exposure in mothers. The large sample size is a key strength of this study, we sampled 8, 586 singleton children and their mothers in this study which accounting for approximately 20% of those preschool children, and almost 80% of total newborns in 1 year in Songjiang district of Shanghai, so study results could be generalized to Songjiang district or other rural areas of Shanghai, China. In this study, the tobacco smoke exposure information, as well as the gestational information including the gestational week and birthweight of children, pre-pregnancy BMI of mother, and the GDM was verified by their birth records in the hospital, which reduced the recall bias in some degree. Meanwhile, potential confounding factors were identified by directed acyclic graphs and subsequently adjusted in logistic regression model is another strength of this study, which ensures a relatively unbiased association between children’s LBW prevalence and mothers’ prenatal tobacco smoke exposure.

This study has some limitations. First, the study design of a cross-sectional study may induce some recall bias and only allow the calculation of prevalence. Second, the detailed information of prenatal first-hand smoke and second-hand smoke exposure among mothers such as daily tobacco consumption, SHS exposure hours were not available, made it impossible to estimate the dose-effect association between prenatal cigarette smoke exposure in mothers and low birthweight in children. Third, the self-reported information of prenatal tobacco smoke exposure among mothers might be under reported and lead to a potential risk of under-estimating the odds ratios between mothers’ prenatal tobacco smoke exposure and LBW in children. So the incorporation of some improvements should be considered in the follow-up studies.

## Conclusions

Children’s LBW is positively associated with mothers’ prenatal tobacco smoke exposure to both FHS and SHS. Therefore, implementing tobacco control measures is crucial to lower smoking prevalence among women, and decrease smoking prevalence of their family members as well as work fellows in Shanghai, China.

## Supplementary information

**Additional file 1.** Questionnaire for prenatal tobacco smoke exposure in mother and low birthweight in their children.

## Data Availability

Data for this study can be made available upon request from the corresponding author. The request should state the title and aim of the research for which the data are being requested.

## Abbreviations

LBW: Low birthweight
FHS: First-hand smoke
SHS: Second-hand smoke
GDM: Gestational diabetes mellitus
CNY: Chinese Yuan
SD: Standard deviation
OR: Odds ratios
CI: Confidence interval
IQR: Interquartile range
GATS: Global adult tobacco survey
DAGs: Directed acyclic graphs
