# Supplementary material for: Low birthweight of children is positively associated with mother’s prenatal tobacco smoke exposure in Shanghai: a cross-sectional study
Source: BMC Pregnancy Childbirth. 2020 Oct 8;20:603. doi: 10.1186/s12884-020-03307-x (PMC7542738; doi:10.1186/s12884-020-03307-x)
Supplement: Supplementary file 1 — Additional file 1. Questionnaire for prenatal tobacco smoke exposure in mother and low birthweight in their children. [file 12884_2020_3307_MOESM1_ESM.docx]

**Prenatal Tobacco Smoke Exposure in Mother and Low birthweight in Their Children**

**Questionnaire**

**(translation version)**

ID :□□-□□□

**Part A: General Information**

A1. how old is your child? _____years

A2. what is the gender of your child? (1) male (2) female

A3. what is your age? _____years

A4. does your child have siblings? (1) yes (2) no

A5. what is your current marital status?

(1) unmarried(single) (2) married (3) divorced (4) others

A6. what is your education level?

1. 0-6 year (illiterate or primary school)
2. 7-9 years (junior high school)
3. 10-12 years (senior high school)
4. >12 years (college and above)

A7. what is the total income in your family last year?

1. <50000 RMB
2. 50000-100000 RMB
3. 100001-150000 RMB
4. 150001-300000 RMB
5. >300000 RMB

A8. are you a local resident? (1) yes (2) no

**Part B: Tobacco Exposure and Perinatal factor**

B1. Have you ever smoked at least one cigarette every day for over six months before or during your pregnancy？ (1) yes (2) no (*if no, please answer question B3*)

B2. Do you still smoke cigarette currently? (1) yes (2) no

B3. Does anyone smoke cigarette around you at home or in workplace for at least 10 minutes each day during your pregnancy? (1) yes (2) no

B4. What is the delivery method of your child? (1) vaginal delivery (2) cesarean delivery

B5. Is your child delivered before the gestational week of 37? (1) yes (2) no

B6. What is your body weight and height before pregnancy ?

body weight______kg, height_____cm

B7. have you been diagnosed as gestational diabetes mellitus (GDM) during the pregnancy ?

(1) yes (2) no

B8. What is the birthweight of your child ? ______g

B9. Is your child admitted to the ICU after the delivery? (1) yes (2) no

B10. How long do you breastfeed your child? ________months

**Part C: Information Extracted from Birth Records**

C1. The body weight of mother before pregnancy. ___________kg

C2. The height of mother before pregnancy._______cm

C3. The gestational week of the newborn. _______weeks

C4. OGTT results at gestational week 24-28.

FBS_______mmol/L, OGTT 1h_____mmol/L, OGTT 2h_____mmol/L,

C5. Does the mother smoke before or during your pregnancy？ (1) yes (2) no

C6. Does the mother exposed to second hand smoke during pregnancy？ (1) yes (2) no

C7. The birthweight of the newborn? ________g

**Part D: Contact information**

D1 what is your home contact phone number? □□□□□□□□

D2 what is your telephone number? □□□□□□□□□□□

D3 Phone number of investigator □□□□□□□□□□□

D4. Investigation date:□□□□-□□-□□

Investigator signature:______________

**母亲孕期烟草暴露与婴儿低出生体重的关系研究**

**问卷调查**

**问卷编号：□□-□□□□**

**A部分：一般信息**

A1. 你孩子现在几岁？_____岁

A2. 你孩子的性别是什么？（1）男 （2）女

A3. 您现在的年龄？_____岁

A4. 你的孩子有兄弟姐妹吗？（1）是 （2）否

A5. 你目前的婚姻状况？（1）未婚（单身） （2）已婚 （3）离婚 （4）其他

A6. 你的文化程度？

（1） 0-6岁（文盲或小学）

（2） 7-9岁（初中）

（3） 10-12岁（高中）

（4） 12年以上（大学及以上）

A7. 你家去年的总收入是多少？

（1） <5万元

（2） 5-10万元

（3） 10-15万元

（4） 15-30万元

（5） >30万元

A8. 你是本地居民吗？（1） 是（2）否

**B部分：烟草暴露与围产期因素情况**

B1. 您是否在怀孕前或怀孕期间每天至少吸一支烟持续6个月以上？

（1）是 （2）否（*如否，请回答问题B3*）

B2. 你现在还抽烟吗？（1）是 （2）否

B3. 在你怀孕期间，在家里或工作场所有没有人每天吸烟至少10分钟？

（1）是 （2）否

B4. 你孩子的分娩方式是什么？（1）阴道分娩 （2）剖宫产

B5. 你的孩子是在孕37周之前分娩的吗？（1）是 （2）否

B6. 你怀孕前的体重和身高是多少？体重_______公斤，身高______厘米

B7. 你在怀孕期间被诊断为妊娠期糖尿病吗？（1） 是（2）否

B8. 你孩子的出生体重是多少？______g(克)

B9. 你的孩子在分娩后住进了重症监护室吗？（1）是 （2）否

B10. 你用母乳喂养孩子多长时间？________月

**C部分：从出生记录中提取的信息**

C1. 母亲怀孕前的体重. ___________公斤

C2. 母亲怀孕前的身高___________厘米

C3. 新生儿的分娩孕周. _______周

C4. 妊娠24-28周的OGTT糖尿病筛查结果.

空腹血糖_____mmol/L; OGTT 1h_____mmol/L; OGTT 2h_____mmol/L;

C5. 产妇在怀孕前或怀孕期间吸烟吗？（1）是 （2）否

C6. 产妇在怀孕前或怀孕期间是否暴露于二手烟？（1）是 （2）否

C7. 新生儿的出生体重？________g

**D部分：联系方式**

D1. 你家的联系电话是什么？□□□□□□□□

D2你的电话号码是多少？□□□□□□□□□□□

D3研究者电话□□□□□□□□□□□□

D4. 调查日期：□□□□-□□-□□

研究者签名：______________
